# P-2027. Care at Home for Remdesivir Treatment of COVID-19: A Mixed Methods Study of Patient and Physician Experiences

**DOI:** 10.1093/ofid/ofae631.2183

**Published:** 2025-01-29

**Authors:** Julia K Nguyen, Rulin C Hechter, Deborah S Ling Grant, Janis F Yao, Cecilia Portugal, Jia-xiao M Shi, Albert Bai Bai, William Towner

**Affiliations:** Kaiser Permanente, Panorama City, California; Kaiser Permanente Southern California Department of Research and Evaluation, Los Angeles, California; Kaiser Permanente Southern California, Pasadena, California; Kaiser Permanente, Panorama City, California; Kaiser Permanente, Panorama City, California; Kaiser Permanente, Panorama City, California; Division of Epidemiologic Research, Pasadena, California; Kaiser Permanente Southern California, Pasadena, California

## Abstract

**Background:**

Hospital in the home and virtual models of care were rapidly implemented during the COVID-19 pandemic. We sought to assess patient and provider perspectives of care at home integrating a three or five-day remdesivir (RDV) treatment regimen in a large integrated health care system in California.

**Figure 1.**
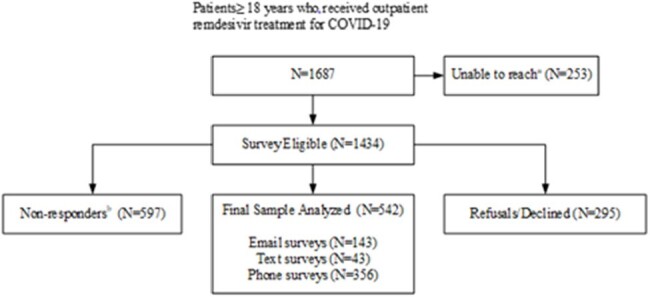
Patient Survey Recruitment Consort Flow Diagram a. Identified as unreachable prior to and during recruitment (deceased, no longer health system members, cognitive impairment, invalid contact information) b. Lost to follow- no response after exhausting all contacts

**Methods:**

A mixed methods study was conducted to assess patient and physician experiences using an online/telephone survey questionnaire developed based on components of the Health Belief Model and Hospital Consumer Assessment of Healthcare Providers. Patients > 18 years of age who received RDV in the outpatient setting (N=1544) and prescribing physicians (N=406) from December 15, 2020-August 31, 2022 were eligible. Descriptive statistics were used to describe categorical responses. A cross-sectional analysis was performed to identify factors associated with patient adherence defined as taking > 80% of prescribed doses and physician adoption of treatment guidelines. Qualitative data were triangulated to determine whether patient/prescriber perceptions about value were validated by health outcomes measured as reduction of COVID-19 related readmission within 30 days of entry into treatment program.

**Figure 2.**
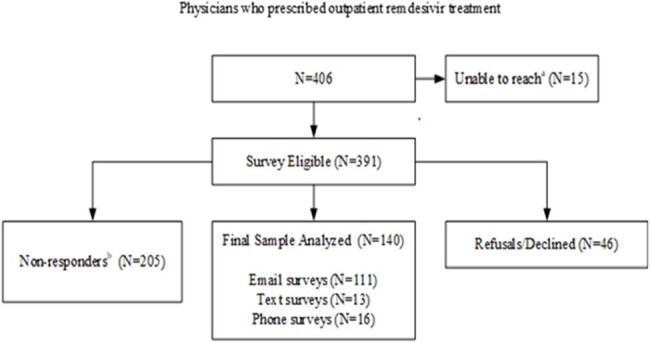
Provider Survey Recruitment Consort Flow Diagram a. No longer health system providers b. Lost to follow-up- no response after exhausting all contacts

**Results:**

Of 540 patient respondents (response rate: 38%, mean age: 58.4 years (SD 16.2), 55.2% male), the majority was of Hispanic ethnicity (48.4.2%), followed by White (32.3%), and Black (12.6%). Of 140 physician respondents (response rate: 34%, mean age: 46.9 years (SD 8.4), 58% male), the majority was of Asian ethnicity (65.8%), followed by White (23%), and Hispanic (7.1%). Adherence was found in 99% of patients having completed > 80% of RDV doses. Adherent patients reported returning to their own home (95.2%), nurses always listened to them (87%), and high health literacy (65%). Feeling knowledgeable was significantly associated with physician prescribing of new unfamiliar medications while under emergency use authorization. (p=0.0007). Patients (80.3%) reporting “Very Satisfied” with medical follow-up were less frequently readmitted within 30 days. (p=0.045)

**Figure:**
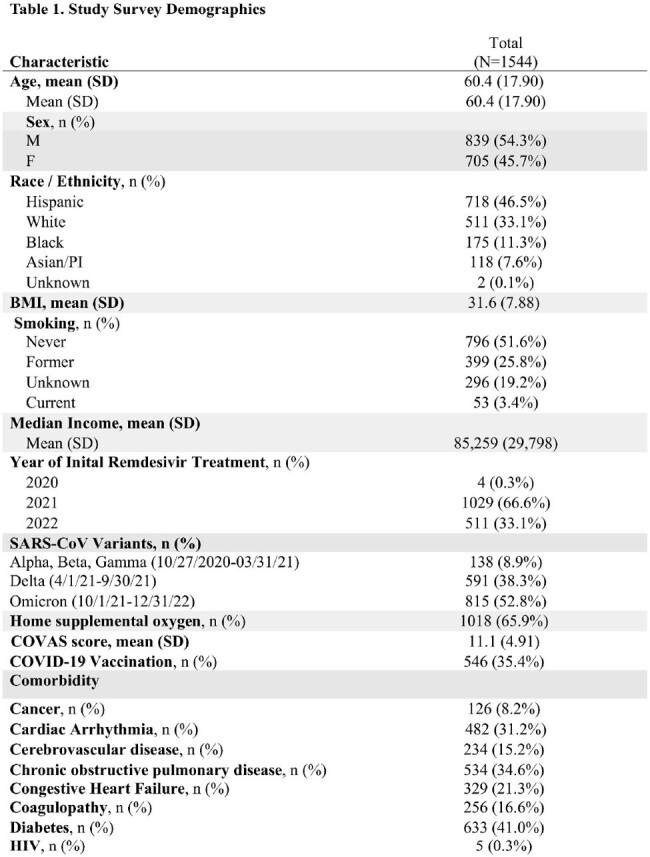

**Conclusion:**

Patients and physicians responded favorably to value-based care at home integrating RDV for treatment of COVID-19. This finding is key to informing future design of alternative modes of health care delivery.

**Figure:**
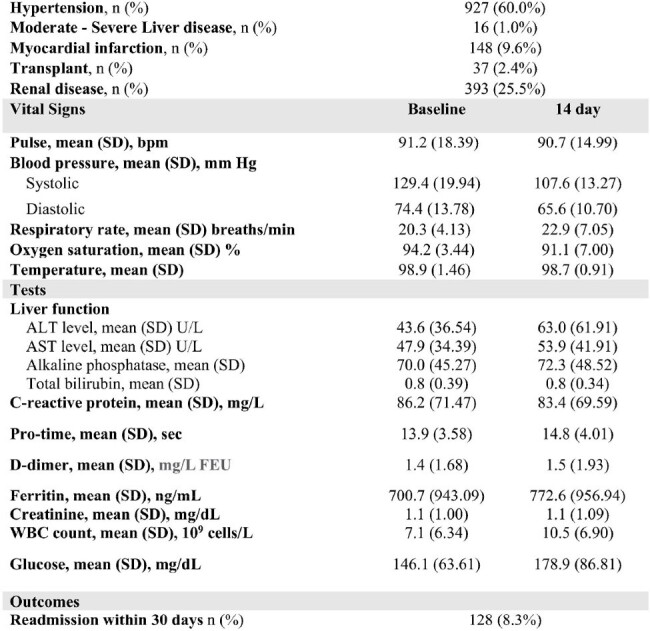

**Disclosures:**

Julia K. Nguyen, PharmD, Gilead Sciences: Grant/Research Support Rulin C. Hechter, MD, PhD, MS, Gilead Sciences: Grant/Research Support William Towner, MD, GSK: Grant/Research Support|Janssen: Grant/Research Support|Moderna: Grant/Research Support|Pfizer: Grant/Research Support

